# The Unique Role of Intravascular Lithotripsy (IVL) in Debulking the Nodular Calcium in Left Main Coronary Artery Bifurcation

**DOI:** 10.7759/cureus.62439

**Published:** 2024-06-15

**Authors:** Dibyasundar Mahanta, Pranjit Deb, Saran Mohanan, Debasis Acharya, Debasish Das

**Affiliations:** 1 Cardiology, SUM Hospital, Bhubaneswar, IND; 2 Cardiology, All India Institute of Medical Sciences, Bhubaneswar, Bhubaneswar, IND

**Keywords:** left main coronary artery, coronary, debulking, nodular calcium, intravascular lithotripsy

## Abstract

Nodular calcium poses a great challenge during coronary intervention. The presence of nodular calcium is associated with poor post-procedural outcomes. Without debulking the nodular calcium, it is extremely difficult to pass the coronary hardwires including the balloons and drug-eluting stents across the lesion. Application of high atmospheric pressure during balloon inflation in the presence of nodular calcium leads to vessel perforation which is a catastrophe during coronary intervention. We report a rare case of nodular calcium in the left main coronary artery bifurcation which was successfully cracked with pulses of intravascular lithotripsy in a 75-year-old male with old anterior wall myocardial infarction. Although rotablation and orbital arthrectomy have a role in modifying calcium nodules in coronary arteries, intravascular lithotripsy was also successful in debulking the nodular calcium in the left main coronary artery bifurcation.

## Introduction

Nodular calcium is the enemy of successful coronary intervention. Nodular calcium hinders the passage of routine balloons and stents across the coronary lesion. Sometimes huge nodular calcium turns the coronary lesion into a balloon undilatable lesion. Fracture of nodular calcium is an essential component in lesion preparation for coronary angioplasty. Dilatation with a noncompliant balloon, scoring balloon or cutting balloon, rotablation, and orbital atherectomy are the modalities for debulking the nodular calcium [1]. We present a rare case where intravascular lithotripsy (IVL) successfully modified the nodular calcium and a good angiographic result was achieved with distal thrombolysis in myocardial infarction (TIMI) III flow. The recent inclusion of IVL in the armamentarium of debulking calcium nodules has virtually changed the scenario of coronary revascularization in highly dense calcium. We performed provisional stenting from the left main coronary artery to the left anterior descending coronary artery across the nodular calcium with good angiographic and clinical outcomes. The present case aims to illustrate the promising role of intravascular lithotripsy in debulking the nodular calcium in coronary arteries which leads to successful coronary revascularization. 

## Case presentation

**Figure 1:**
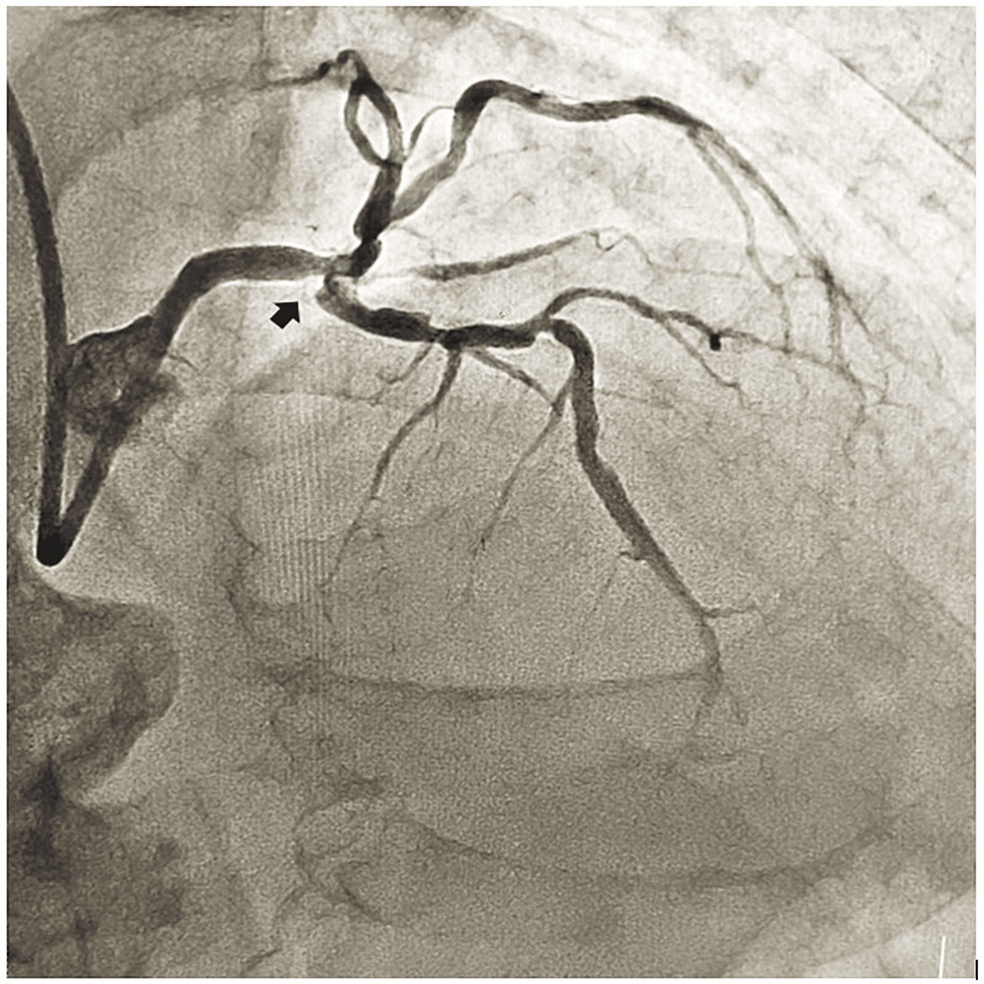
Nodular calcium in the left main coronary artery bifurcation

An octogenarian male patient presented to the cardiology outpatient department of All India Institute of Medical Sciences (AIIMS), Bhubaneswar, India in January 2024 with rest angina in the last three days with diaphoresis and shortness of breath. He was nondiabetic, nonhypertensive, and a smoker for the last 30 years. During clinical examination, he had a heart rate of 80 beats per minute with a blood pressure of 110/80 mm Hg in the right arm supine position. His cardiovascular system auscultation revealed the presence of left ventricular fourth heart sound. His electrocardiogram (ECG) revealed the presence of a QS pattern across anterior precordial leads. His echocardiography revealed the presence of regional wall motion abnormality in the left anterior descending coronary artery territory with moderately impaired left ventricular systolic function (EF=37%). His serum Troponin T was within the limit. He was subjected to a right transradial coronary angiogram which revealed a large nodular calcium in the left main coronary artery bifurcation (Figure 1) causing subtotal critical occlusion.

**Figure 2:**
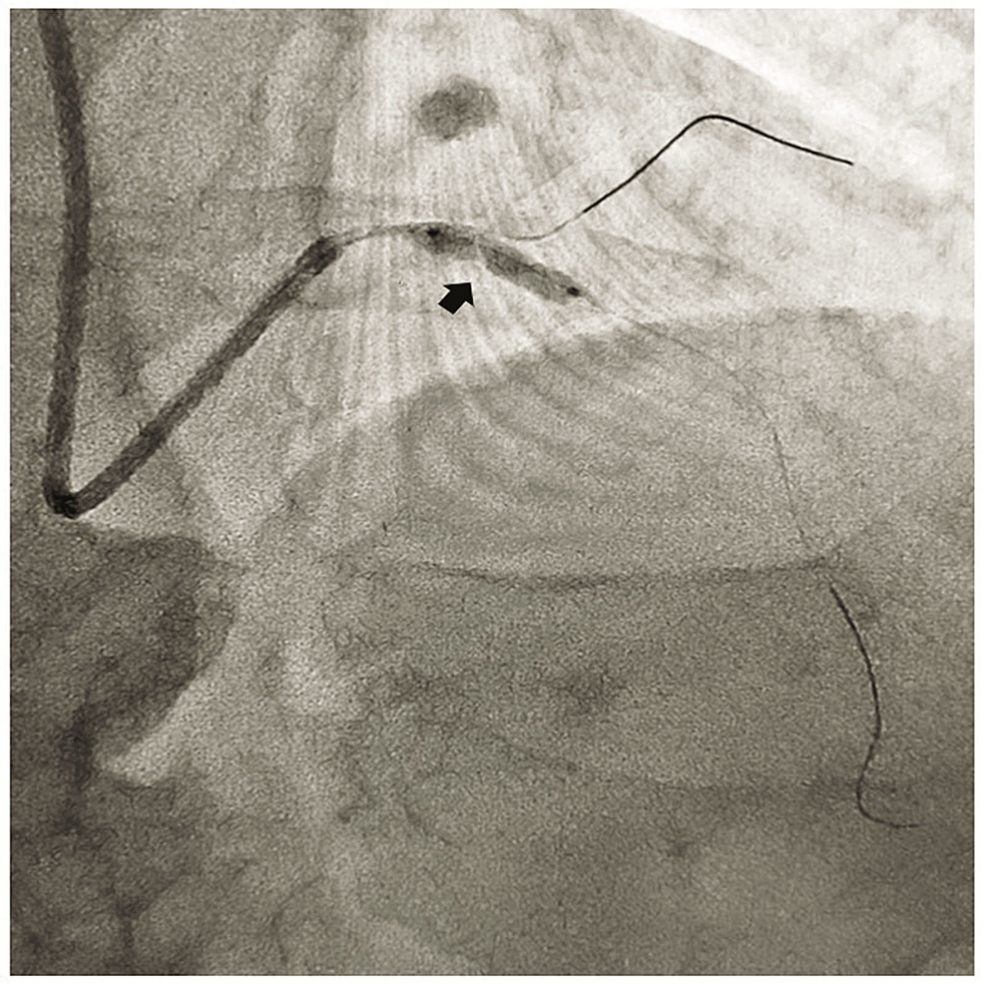
3x12 mm shock wave C2 balloon across the nodular calcium

It was decided to crack the nodular calcium with a scoring balloon. The left main coronary artery was engaged with an extra backup guide catheter 6F 3.5 and the lesion in the left anterior descending coronary artery was crossed with 0.014 '' Fielder FC coronary guide wire and a 0.014'' coronary guide wire was parked in the left circumflex coronary artery for protection of the side branch. Initially, we dilated the osteoproximal coronary lesion with a 2.5x10 mm scoring balloon which did not yield the lesion. Then it was decided to debulk the osteoproximal nodular calcium in the left main coronary bifurcation with IVL. Then a 3.0 × 12-mm Shockwave C2 (Shockwave Medical) IVL balloon was placed across the nodular calcium (Figure 2) and five cycles of 10 pulses (a total of 50 pulses out of 80 pulses) were delivered over the nodular calcium after which the waist of the shock wave balloon completely disappeared. We did not deliver the full 80 shock wave pulses as the patient developed bradyarrhythmia with significant hypotension and severe angina after delivering 50 shock wave pulses.

**Figure 3:**
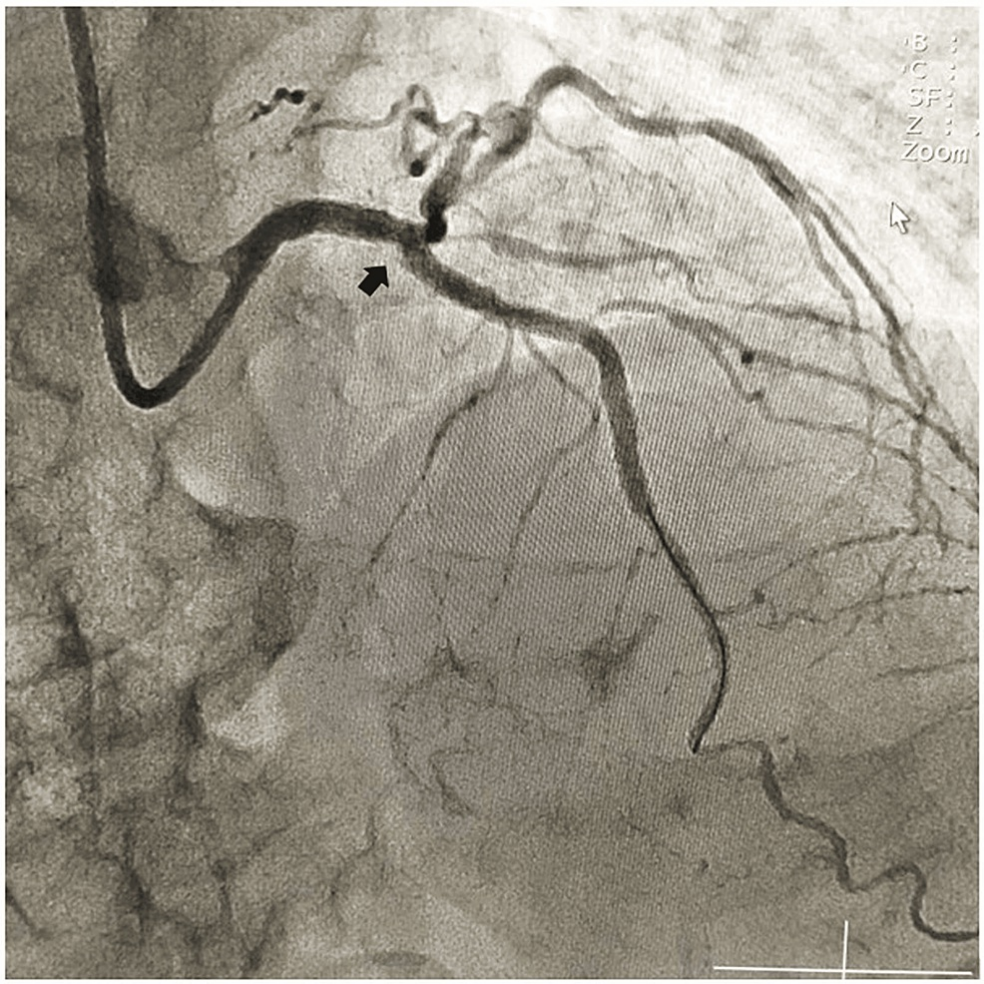
Good angiographic result with distal thrombolysis in myocardial infarction (TIMI) III flow

We provisionally deployed a 3x24 mm drug-eluting stent (DES) across the left main coronary artery to the left anterior descending coronary artery at 14 atm pressure. Post-deployment, a lesion in the distal left anterior descending coronary artery became evident for which another 2.75 mmx16 mm DES was deployed across the lesion with good angiographic results with distal TIMI III flow (Figure 3).

Post-procedure, the patient developed hypotension with minimal pericardial effusion which resolved in the next 72 hours. The patient was discharged in hemodynamically stable condition and was doing well in follow-up after one month. We did not opt for rotational atherectomy in the aforesaid patient due to 90-degree angulation from the left main coronary artery to the left anterior descending coronary artery although rotablation is another modality in debulking the nodular calcium. IVL was able to debulk the nodular calcium resulting in good angiographic results with good clinical outcomes in the octogenarian.

## Discussion

Nodular calcium in the coronary artery is an enemy of successful coronary intervention. Nodular calcium obstructs the passage of the routine coronary balloons and stents across the coronary artery. Nodular calcium acts as a solid rock inside the coronary artery and also prevents imaging. We could not perform coronary imaging in the aforesaid case as an intravascular ultrasound catheter could not be negotiated across the lesion due to the presence of nodular calcium. Lesions with large nodular calcium often present with chronic stable angina. Rotablation, cutting balloons, scoring balloons and IVL are the current modalities used in debulking of the nodular calcium. Shaking and shaving are the two maneuvers intended to treat the coronary calcium nodules. Shaking principally refers to the use of IVL whereas shaving refers to the use of rotational arthrectomy during coronary angioplasty. A calcified nodule constitutes 12% of calcified coronary stenosis [2]. The presence of a calcified nodule presents a risk factor for poor stent expansion [3] and serves as an independent risk factor for major adverse cardiac outcomes over time [4]. IVL is safe and effective in debulking nodular calcium as per the DISRUPT-CAD III pooled data analysis [5]. The use of IVL is a relatively safer option as compared to rotational atherectomy in coronary ostial lesions. As the index patient had coronary ostial nodular calcium and the patient was an octagenarian, we decided to debulk the nodular calcium with IVL rather than shaving with rotational arthrectomy. Scoring balloons in the index case could not yield the nodular calcium and it was impossible to pass coronary hard wires across the nodular calcium. Calcium nodules are most often present with diffuse vessel calcification; hence it is extremely important to debulk the whole coronary segment as compared to the calcium nodule only during lesion preparation. Nodular calcium presents the tip of the iceberg of calcified atherosclerosis. The index patient had extensive calcification up to the distal left anterior descending coronary artery which was predilated with a non-compliant balloon with subsequent stenting with a DES with a good angiographic result and distal TIMI III flow. The advantages of IVL are that it is simple, effective, and less time-consuming in debulking the nodular calcium. Rare cases of distal slow flow, ventricular tachyarrhythmia, and vessel rupture have been reported with IVL. Adequate deairing of the IVL balloon is mandatory before its inflation across the calcified lesion. The main disadvantage of the use of IVL balloons is that most often patients are not able to bear the cost of the procedure which is more as compared to rotablation. The principal limitation of the IVL balloon is that it cannot be crossed easily across heavily calcified balloon un-dilatable lesions because of its larger profile. IVL safely and effectively cracks the nodular calcium in critical coronary regions. 

## Conclusions

We report a rare case of nodular calcium in the left main coronary artery bifurcation which was successfully treated with a DES after debulking with IVL. Although many modalities including scoring balloons, cutting balloons, excimer laser, and rotational and orbital atherectomy are the modalities to treat the nodular calcium, IVL is also a safe and effective tool in debulking the nodular calcium in critical regions. 
